# Scientific

**Published:** 1889-12

**Authors:** 


### SCIENTIFIC ITEMS.

There is a class of vegetables that mix badly when in bloom, and which in one season become almost worthless. We allude to the various vines. Farmers and gardeners do not, as a rule, exercise enough care in planting them. Take, for instance, the several varieties of squashes. They should be planted quite a distance apart. Last autumn, while attending a fair, we noticed an exhibit of squashes marked Hubbards, which were yellow, showing that they had been planted too near the Marrow, as they had all the characteristics of that variety. Squashes should never be planted near pumpkins, watermelons near citrons, or cucumbers near muskmelons. If so planted they will in one season become worthless hybrids. Too much care could not be exercised in this matter, and the farmer should give careful attention to planting, or the whole crop may be a loss.—*Ex.*

Many people have heard of the singing beach at Manchester, Mass., where the sand, when driven over or stirred, gives out a peculiar and not unmusical sound; but few, very few, people know that near Pescadero, Cal., a beach exists much larger, giving out sounds in no uncertain manner. The beach near Manchester is said to be one-fifth of a mile in extent, but at Pescadero an investigating party found the sounds very clear, though varying in loudness, for a distance of one and a-half miles along the coast-line. The sound is loudest and most distinct where the sand is dry on top and damp beneath. A light vehicle driven over it gives a clear, musical sound, a footstep not quite so loud, and even the hands or a stick stirring or lifting the sand causes it to "sing" quite plainly.—*Ex.*

Every one who has looked through a large telescope has noticed the purple rim around the edge of the object viewed. This is known as the secondary chromatic aberration, and has been regarded as a serious defect by astronomers. The announcement is made that Professor C. S. Hastings, of the Sheffield Scientific School of Yale College, has succeeded in combining two Abbe glasses in such a way as to eliminate entirely this aberration, and to reveal objects in their natural colors. The discovery is one of substantial value—especially seeing that it can be used for celestial photography—and will doubtless greatly facilitate astronomical investigation.—*Zion's Herald.*

Three new asteroids have recently been added to the system by Palisa at Vienna. The new planets are all extremely small: of the eleventh or twelve magnitude.—*Ib.*

If a current of electricity be passed through a coil of wire with a hole through its centre, and a small iron or wire be brought near it, it is drawn into the coil with considerable force, and if the current be stopped at the right instant, the rod may be made to jump entirely through the coil. A recent invention, which is based on this principle, consists of a small rail road, slightly raised above the ground, and surrounded at frequent intervals by coils of wire. A hollow steel carriage runs upon the rails and passes through the coils, the electric current being automatically sent through each coil successively at the proper moment. The steel carriage, which represents the iron rod in the original experiment, is drawn through the coils with great force, and it is claimed that a speed of one hundred miles can be easily obtained. Letters and small packages could be thus sent from Boston to New York in two hours, and to Chicago in half a day. On theoretical grounds there would seem to be no reason why the idea is not a practical one, and if the cost of constructing and operating the new style of rail road is not too much to prevent its use, the invention will be a very useful and important one.—*Pop. Sci. News.*

According to Miss Eva M. A. Bewsher of Mauritius, it is a well authenticated fact that each hive in tropical countries has its "ventilating bees" during the hot season. Two or three of these bees are stationed at the entrance of the hive and cool the interior by incessant fanning with their wings. They are relieved at intervals by others, and while on duty are kept constantly at work by a sort of patrol of bees.—*Ex.*

Some years ago the greenish color of some of the sloths was attributed to the presence of an alga upon the hair. Madam Weber von Bosse has recently described two genera, and three species of these parasite plants. The one new genus is green; the other with its two species is violet. From 150,000 to 200,000 individuals of these algæ may occur upon a single hair.—*Ib.*

**To Preserve Flowers.**—Ladies who surround the stems of their corsage boquets with moistened powdered willow charcoal, which may in turn be wrapped in moss or cotton, will find their flowers remaining fresh long after the departure of all beauty from those of their less thoughtful neighbors. The same substance placed in the bottom of the vase in which flowers are kept will be very useful, provided the stems are cut off with a sharp knife once or twice a day.

**Protect Plants from Frost.**—A successful amateur says she does n't allow frost bites to her plants so long as she has a two gallon jug in the house, and can get up hot water. She first coats the jug with layer after layer of paper to keep in the heat and also to modify it. Then, in severe nights she places this jug on her centre table, and around it the tender plants, and over all a sheet of water proof cloak, supported by some sticks thrust into the pots. This method carries the plants safely through any kind of a cold night.

